# Testing the disconnectome symptom discoverer model on out-of-sample post-stroke language outcomes

**DOI:** 10.1093/brain/awad352

**Published:** 2023-10-11

**Authors:** Thomas M H Hope, Douglas Neville, Lia Talozzi, Chris Foulon, Stephanie J Forkel, Michel Thiebaut de Schotten, Cathy J Price

**Affiliations:** Wellcome Centre for Human Neuroimaging, Department of Imaging Neuroscience, Institute of Neurology, University College London, London WC1N 3AR, UK; Department of Psychological and Social Sciences, John Cabot University, 00162 Rome, Italy; Wellcome Centre for Human Neuroimaging, Department of Imaging Neuroscience, Institute of Neurology, University College London, London WC1N 3AR, UK; Department of Neurology and Neurological Sciences, Stanford University School of Medicine, Stanford, CA 94304, USA; Brain Connectivity and Behaviour Laboratory, Sorbonne Universities, Paris 75006, France; Queen Square Institute of Neurology, University College London, London WC1N 3BG, UK; Brain Connectivity and Behaviour Laboratory, Sorbonne Universities, Paris 75006, France; Queen Square Institute of Neurology, University College London, London WC1N 3BG, UK; Donders Institute for Brain Cognition Behaviour, Radboud University, Nijmegen 6525, The Netherlands; Centre for Neuroimaging Sciences, Department of Neuroimaging, Institute of Psychiatry, Psychology and Neuroscience, King’s College London, London SE5 8AF, UK; Brain Connectivity and Behaviour Laboratory, Sorbonne Universities, Paris 75006, France; Groupe d'Imagerie Neurofonctionnelle, Institut des Maladies Neurodégénératives-UMR 5293, CNRS, CEA, University of Bordeaux, Bordeaux, 33076, France; Wellcome Centre for Human Neuroimaging, Department of Imaging Neuroscience, Institute of Neurology, University College London, London WC1N 3AR, UK

## Introduction

Stroke is common, and its consequent brain damage can cause various cognitive impairments. Associations between where and how much brain lesion damage a patient has suffered, and the particular impairments that injury has caused (lesion-symptom associations) offer potentially compelling insights into how the brain implements cognition.^1^ A better understanding of those associations can also fill a gap in current stroke medicine by helping us to predict how individual patients might recover from post-stroke impairments.^2^ Most recent work in this area employs machine learning models trained with data from stroke patients whose mid-to-long-term outcomes are known.^2-4^ These machine learning models are tested by predicting new outcomes—typically scores on standardized tests of post-stroke impairment—for patients whose data were not used to train the model. Traditionally, these validation results have been shared in peer-reviewed publications describing the model and its training. But recently, and for the first time in this field (as far as we know), one of these pre-trained models has been made public—The Disconnectome Symptom Discoverer model (DSD) which draws its predictors from structural disconnection information inferred from stroke patients’ brain MRI.^5^

Here, we test the DSD model on wholly independent data, never seen by the model authors, before they published it. Specifically, we test whether its predictive performance is just as accurate as (i.e. not significantly worse than) that reported in the original (Washington University) dataset, when predicting new patients’ outcomes at a similar time post-stroke (∼1 year post-stroke) and also in another independent sample tested later (5+ years) post-stroke. A failure to generalize the DSD model occurs if it performs significantly better in the Washington data than in our data from patients tested at a similar time point (∼1 year post-stroke). In addition, a significant decrease in predictive performance for the more chronic sample would be evidence that lesion-symptom associations differ at ∼1 year post-stroke and >5 years post-stroke.

## Materials and methods

### The Disconnectome Symptom Discoverer model

The technical details of the DSD model are described in Talozzi *et al*.,^5^ though most of those details are irrelevant to this work, where we treat the model as a black box.

The input to the DSD model is a binary lesion image in standard Montreal Neurological Institute (MNI) space. The outputs are predicted scores on a wide range of tasks, which include tasks assessing language outcomes after stroke, derived from the Western Aphasia Battery (WAB).^6^

### Test data

Our independent sample is drawn from the Predicting Language Outcomes and Recovery After Stroke (PLORAS) database.^7^ The database associates more than 1000 stroke patients’ brain MRI and demographic/clinical data with language scores derived from the Comprehensive Aphasia Test (CAT).^8^ Though the CAT is similar to the WAB in that both purport to measure the same or similar language skills, CAT tasks are not identical to WAB tasks. For this reason, we focused on two CAT tasks only. The CAT semantic fluency task was selected because it is essentially the same in the WAB and the CAT—asking patients to name as many animals as possible in 1 min. Likewise, the visual naming task is similar in the WAB and CAT—requiring patients to name a series of common objects, depicted in pictures.

MRI preprocessing steps are described in detail elsewhere^7^; we use a semi-automated procedure to segment lesions, which is an elaboration of the popular Unified Segmentation algorithm, extended to cater to the damaged brain.^9^ This is in contrast to the Washington dataset, in which lesions were segmented by hand. Still, both approaches yield the required inputs to the DSD model, which are a 2 × 2 × 2 mm binary lesion image per patient, in standard MNI space. The DSD models converts these lesion images into a disconnectome map using routines distributed with the BCBtoolkit, and making reference to a dataset of *n =* 176 tractographies derived from 7 T MRI diffusion-weighted scans from healthy controls.

### Analyses

We report the results of two types of analysis. The first is a simple generalization of the pre-trained DSD model to two subsamples of PLORAS patients. The first sample, ‘PLORAS 1 year’ (*n* = 314), includes patients whose naming and fluency skills were measured between 6- and 18-months post-stroke: i.e. near the 1-year post-stroke time point at which outcomes were assessed in the Washington dataset. The second sample includes PLORAS patients whose naming and fluency skills were assessed >5 years post-stroke, which we refer to as the ‘PLORAS 5+ years’ sample (*n* = 340). The rationale here follows from increasing evidence (including that derived from PLORAS data) that recovery from aphasia (language impairments) after stroke can continue over years.^10^ To the extent that this is true, the DSD model’s predictive performance should be better when predicting outcomes at ∼1 year post-stroke (for which the model was trained) than when predicting outcomes many years later.

Since the DSD model does not predict CAT scores directly, we measure the quality of the predictions as the correlation between the predictions and selected CAT scores, and compare them to those reported for the Washington dataset (Supplementary Table 2 in Talozzi *et al*.^5^), using a Fisher *r*-to-*z* transform. We also report comparisons between the DSD model’s performance on PLORAS 5+ years patients and: (i) reported performance on the Washington dataset; and (ii) empirical predictive performance on the PLORAS 1 year patients.

## Results

Table 1 reports summary statistics for the patient samples. When predicting outcomes for the PLORAS 1 year sample, performance was not significantly different from that reported for the Washington dataset (used to test the model in the report than introduced it). However, performance was significantly worse when predicting outcomes for the PLORAS 5+ years sample (though only marginally, for the fluency score) (Table 2).

**Table 1:** Summary statistics for two subsamples of PLORAS patients

Variable name	PLORAS 1 year (median/IQR)	PLORAS 5+ years (median/IQR)
Time post-stroke, years	0.98/0.45	7.98/5.70
Age at stroke onset, years	59.38/18.55	53.64/18.11
Semantic fluency score	14.0/10.0 (109 impaired)	12.5/10.0 (137 impaired)
Naming score	45/9 (73 impaired)	46/8 (91 impaired)
Sample size	314	340
Lesion volume, cm^3^	2.02/12.06	18.40/38.10

**Table 2:** Predictive results, and comparisons between them

	*R* (predicted versus empirical)/*P*			*Z* (comparing *R*’s)/*P*		
	Washington	PLORAS 1 year	PLORAS 5+ years	Washington versus 1 year	Washington versus 5+ years	PLORAS 1 year versus 5+ years
Naming	0.35/0.024^a^	0.31/<0.001	0.00/0.86	0.28/0.78	2.92/0.004	4.27/<0.001
Fluency	0.41/0.003^a^	0.34/<0.001	0.19/<0.001	0.65/0.52	1.94/0.053	2.07/0.04

The leftmost three columns report the correlation between predicted scores and empirical scores for both naming (first row) and fluency (second row).

^a^Inferred from the report of the DSD model (Supplementary Table 2 in Talozzi *et al*.^5^), rather than calculated directly.

## Discussion

Our results suggest that the DSD model generalizes out-of-sample to patients not seen by the model’s authors before they released it. Some numerical loss of predictive performance was observed for both fluency and naming skills of PLORAS patients assessed ∼1-year post-stroke, but this was not significant for either outcome variable. This is a good result for the model, and also a confirmation that CAT scores measuring naming and fluency skills are at least somewhat related to the WAB scores that purport to measure the same skills. Predictive performance was worse for the PLORAS 5+ years dataset, both relative to the Washington dataset (where the difference was only significant for naming), and relative to the PLORAS 1 year dataset (where the differences were significant for both naming and fluency). This might be evidence that lesion-symptom associations change between 1 and 5 or more years post-stroke, consistent with other reports from the same database.^10^ However, the PLORAS 5+ year patients also had significantly larger lesions than the PLORAS 1-year sample (Wilcoxon rank sum test: *z* = 10.99, *P* < 0.001), which could also explain the difference.

One limitation of this work is that MRI scans were acquired contemporaneously with behavioural data in the PLORAS dataset, whereas in the Washington dataset the model is predicting behavioural scores at ∼1 year post-stroke from scans acquired acutely, within 2 weeks post-onset. This difference is noteworthy, because stroke-induced lesions can change over time post-stroke.^2^ The difference might explain the numerical (non-significant) performance loss for the DSD model when predicting PLORAS 1 year patients’ fluency and naming skills. Conversely, it might confound the comparison between performance on the Washington and PLORAS 5+ years datasets. The comparison could also be confounded because PLORAS patients’ lesions were segmented algorithmically, whereas Washington patients’ lesions were segmented manually.

Importantly at first sight the DSD model’s performance appears not to match previously reported results in the field. For example, some of us have previously reported predictions that explain ∼60% of the variance in fluency and naming skills,^3^ whereas the DSD model’s predictions explain <20% of that variance. The comparison may be misleading because the prior results are derived from (i) models combining lesion data with non-lesion factors; and (ii) internal cross-validation, rather than genuinely out-of-sample testing, as used here. It remains to be seen whether those previously reported performance levels can be replicated in analyses like that presented here.

Despite these limitations, our results are positive because we observe the DSD model to perform roughly as well in PLORAS patients as in the model authors’ own data, despite the differences between the two datasets. That this equivalence wanes when predicting the same skills many years after stroke, highlights the need to model changes in language skills over many years post-stroke. As far as we know, this is the first time that a pre-trained post-stroke prognostic model has been validated in this way, and in this sense, the DSD model represents a definite advance for the field. We hope that others will consider taking this same step in future, in releasing their own pre-trained models.

## Data Availability

PLORAS test data, used in this work, can be made available to readers on reasonable request. For the purpose of Open Access, the author has applied a CC BY public copyright licence to any Author Accepted Manuscript version arising from this submission.
